# Activation of Piezo1 and TRPV4 channels contributes to hCMEC/D3 cell mechano-sensing

**DOI:** 10.3389/fphys.2025.1633251

**Published:** 2025-09-08

**Authors:** Wenhan An, Min Sun, Xiulin Zhang, Laigang Huang, Hanwen Liu, Baojuan Cui

**Affiliations:** ^1^ Department of Rehabilitation Medicine, The Second Hospital of Shandong University, Jinan, China; ^2^ Department of Rehabilitation Medicine, The Affiliated Yantai Yuhuangding Hospital of Qingdao University, Yantai, China; ^3^ Department of Urology, The Second Hospital of Shandong University, Jinan, China

**Keywords:** ATP, human brain microvascular endothelial cells, mechanosensory transduction, Piezo1 channel, TRPV4 channel

## Abstract

**Introduction:**

TRPV4 and Piezo1 channels have been considered two important mechanical sensors. To examine the role of these two channels in blood brain barrier (BBB) mechano-sensing, the expression and function of TRPV4 and PIEZO1 in hCMEC/D3, an *in vitro* model for human BBB endothelial cells, were investigated.

**Methods:**

TRPV4 and PIEZO1 mRNA and protein expression were analysed by RT-PCR, immunofluorescence and western blot, respectively, while their mechano-sensing function was investigated by calcium imaging.

**Results:**

Among the four mechano-sensing channels examined (TRPV2, TRPV4, PIEZO1 and Piezo2), TRPV4 and PIEZO1 were the two most abundantly expressed in hCMEC/D3 cells at the mRNA level. TRPV4 and PIEZO1 proteins were detected by immunofluorescence, western blot. The calcium imaging using channel-specific agonists/antagonists provided evidence of their function. Mechanical stimuli (poke or flow shear stress) induced a prominent increase in intracellular Ca^2+^([Ca^2+^]_i_) in hCMEC/D3 cells, which was significantly inhibited by application of selective TRPV4 or Piezo1 antagonists. Activation of PIEZO1 and TRPV4 using selective agonists resulted in the release of adenosine triphosphate (ATP) but not nitric oxide (NO). Extracellular ATP hydrolysis with apyrase, or blocking of P2X and P2Y purinoceptors with PPADS, significantly reduced mechanical stimulus-induced increases in [Ca^2+^]_i_ in both the stimulated cell and neighboring cells.

## Introduction

The blood-brain barrier (BBB) is a highly selective lipophilic barrier located between the systemic circulation and brain tissue (Abbott, 2005); it is the largest interface for blood-brain exchange and has important roles in maintaining central nervous system (CNS) homeostasis. The BBB is mainly composed of human brain microvascular endothelial cells (HBMECs), pericytes, an astrocytic capillary layer surrounding the endothelium, and the basement membrane (Sweeney et al., 2019). HBMECs are important for BBB morphology, and at least 85% of the BBB surface area is derived from these cells, which have crucial functions in controlling BBB permeability and regulating brain microcirculation. Further, HBMECs have unique morphological, structural, and functional features that distinguish them from endothelial cells situated elsewhere in the body (Daneman and Prat, 2015).

The BBB can respond to various mechanical cues, such as blood flow induced shear stress (SS), pressure, and substrate stiffness (Choublier et al., 2022; Weksler et al., 2013; Yan et al., 2023). HBMECs detect and respond to this mechanical force by triggering several downstream biochemical signaling pathways that control different cellular properties under physiological and pathophysiological conditions (Fang et al., 2019). Thus, mechanical force on HBMECs has a key role in maintaining the structural and functional integrity of the BBB (Garcia-Polite et al., 2017; Wang et al., 2020); however, the molecular mechanism underlying BMEC sensing of mechanical force remains elusive.

Transient receptor potential (TRP) channels are important sensors of cellular responses to mechanical and chemical stimuli and temperature changes in various cell types. Among all the TRP channels, TRPV4 is considered a mechanical sensor, and the role of TRPV4 in sensing SS has been demonstrated in vascular endothelial cells (Li et al., 2015; Sonkusare et al., 2012; Sonkusare et al., 2014; Yang et al., 2020). For example, blood flow SS induces TRPV4 activation in endothelial cells, leading to carotid diastole, an effect that can be blocked using a selective TRPV4 antagonist. TRPV4 channels are abundantly expressed in the human BBB (Luo et al., 2021), and have a critical regulatory role in human BBB integrity (Kumar et al., 2020; Luo et al., 2021; Luo et al., 2020; Michinaga et al., 2021; Rosenkranz et al., 2020). TRPV4 expression in rat or human HBMECs has also been demonstrated (Berra-Romani et al., 2019; Luo et al., 2020); however, its role in mechano-transduction in HBMECs has yet to be examined.

Piezo1 and Piezo2 are mammalian mechano-transduction ion channels whose roles in various cell types, including endothelial cells, have been extensively studied in recent years (Beech and Kalli, 2019; Coste et al., 2012; Delmas et al., 2022; Douguet et al., 2019; Wang et al., 2022). In addition to sensing mechanical force, Piezo1 channels can be activated by the chemical agonist compound, Yoda1 (Botello-Smith et al., 2019); however, no specific agonist of Piezo2 has been discovered. Piezo1 channels mediate transient Ca^2+^ release in arterial endothelial cells and mice capillary endothelial cells in response to mechanical stimuli, such as SS (Beech and Kalli, 2019); a recent study showed that Piezo1 channels act as mechanosensors in central nervous system capillaries in mice (Harraz et al., 2022). However, to our knowledge, no study has investigated the expression and function of PIEZO1 channels in human HBMECs.

The immortalized human brain microvascular endothelial cell line, hCMEC/D3, exhibits key features of the BBB, has been proposed as a suitable *in vitro* model for BBB endothelial cells (Weksler et al., 2013), and is the most widely-used and best-characterized human BMEC cell line. In the present study, expression of TRPV4 and PIEZO1 channels in hCMEC/D3 cells at the mRNA, protein, and functional levels were examined using RT-PCR, immunohistochemistry, and Ca^2+^ imaging approaches, respectively. Most importantly, their roles in hCMEC/D3 mechano-sensing were also investigated.

## Materials and methods

### Cell preparation

The hCMEC/D3 cell line was obtained from Jennio Bio (Guangzhou, China). Cells were plated in cell culture flasks, incubated in a 5% CO_2_ atmosphere at 37 °C, and culture medium (90% Dulbecco’s Modified Eagle Medium High Glucose + 10% fetal bovine serum; Gibco) changed every 2 days. Cells were used for experiments at passage 2–8. For intracellular Ca^2+^ [Ca^2+^]_i_ measurement and immunofluorescence experiments, cells were plated onto glass coverslips (8 mm diameter) and grown to 80% and 50% confluence, respectively.

### Single cell mechanical stimulation by poke

Cells on glass coverslips were placed in a chamber perfused with normal Hanks’ buffered saline solution (HBSS) containing (in mM): 138 NaCl, 5 KCl, 0.3 KH_2_PO_4_, 4 NaHCO_3_, 2 CaCl_2_, 1 MgCl_2_, 10 HEPES, and 5.6 glucose (pH 7.4). Mechanical stimulation of single cells was performed as described by Neuhaus et al. (2020). To mechanically stimulate single cells, glass micropipettes with closed and rounded tips (approximately 2 μm) were used to deflect the plasma membrane. Glass micropipette movement was controlled using a motorized MP-285 micromanipulator (Sutter Instruments, Novato, CA, United States); when the tip was close to the cell, the micropipette was dropped in 2 μm increments, to induce membrane deflection.

### Mechanical stimulation by flow SS

**FIGURE 1:**
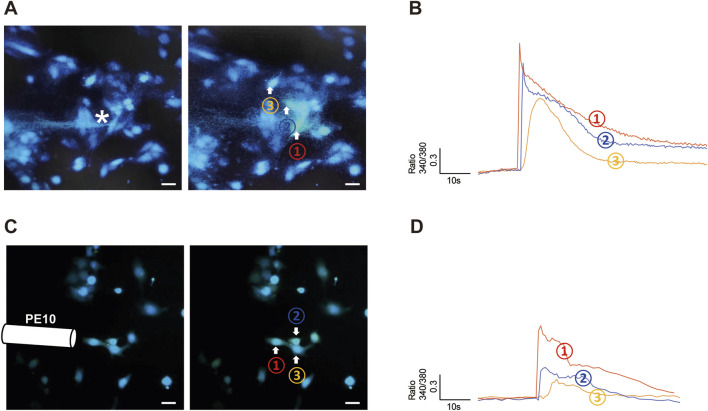
Poke and flow shear stress evoked an [Ca^2+^]_i_ increase in hCMEC/D3 cells. **(A,B)** A single hCMEC/D3 cell (Cell 1) was stimulated with a finely closed round glass micropipette tip (indicated by the star), which evoked an increase in [Ca^2+^]_i_ in the stimulated cell and in surrounding cells (cells 2 and 3). The amplitude of the [Ca^2+^]_i_ increase reduced with distance from the stimulated cell (that in cell 2 was greater than that in cell 3). **(C,D)** Flow shear stress (SS) stimulation evoked an [Ca^2+^]_i_ increase in a group of hCMEC/D3 cells. SS was administered by changing the flow rate from 2 to 20 mL/min. [Ca^2+^]_i_ increase is expressed as the ratio of fluorescence at 340 and 380 nm (F340/F380). Scale bar = 20 μm.

Cells on glass coverslips were perfused with a PE10 tube filled with HBSS. One end of the PE10 tube was connected to a 50 mL syringe pump (Genie touch, Kent Scientific Co.) and the other end was mounted on the probe of a patch-clamp recording platform (EPC10, HEKA Electronik, Lambrecht/Pfalz). The probe approached the cells at a 45° angle, controlled using a MP-285 micromanipulator (Sutter Instrument) and with the tip of the PE10 tube 30–50 μm away from the stimulated cells (Figure 1C). Flow SS was induced by changing the perfusion flow rate from 2 to 20 mL/min.

### Ca^2+^ imaging

Ca^2+^ imaging experiment was conducted as described in our previous study (Wen et al., 2018). In brief, hCMEC/D3 cells on glass coverslips (n = 50–60 cells) were loaded with 1 µM fura-2/AM for 30–45 min at 37 °C in normal HBSS. Subsequently, cells were washed with HBSS to remove excessive external fura-2 dye. The glass coverslip was then mounted on a stage equipped with an inverted fluorescence microscope and HCMEC/D3 cells continuously perfused with HBSS driven by gravity. Cells were exposed to alternating 340- and 380-nm wavelength (bandwidth, 10 nm) ultraviolet light (1/s). Excitation time and image acquisition were controlled using Meta Fluor R (Molecular Devices, Sunnyvale, CA, United States). Data were analyzed using program C-Imaging (Compix). Background was subtracted to minimize camera dark noise. The ratio of fluorescence signal measured at 340 nm divided by the fluorescence signal measured at 380 nm was used to measure the increase of intracellular Ca^2+^. Baseline intracellular Ca^2+^ concentration was determined from the average of five to eight measurements obtained before drug application. Amplitudes of peak Ca^2+^ responses were computed as the difference between the peak value and the baseline value (Δ340/380). A significant increase in [Ca^2+^]_i_ was considered if the ratio changed by >0.1.

### RT-qPCR

Total RNA was extracted from cultured cells using an RNA Simple Total RNA kit (YISHAN, Shanghai, China), according to the manufacturer’s instructions. An ultraviolet spectrophotometer was used to determine RNA concentrations. Reverse transcription was conducted using 1 µg RNA with the HiScript III RT SuperMix kit (Vazyme, Nanjing, China), and complimentary-DNA was used for RT-qPCR amplification. TRP and Piezo1 channel mRNA levels were measured by qPCR using SYBR qPCR Master Mix (Vazyme) and a QuantStudio™ 5 system (Thermo Fisher, Waltham, MA, United States), with the following cycling conditions: initial denaturation at 95 °C (2 min) followed by 40 cycles of denaturation at 95 °C (15 s) and combined annealing and extension at 60 °C (30 s). Primers for TRP and Piezo channels were generated by Sangon Biotech (Shanghai, China); the sequences are shown in Table 1.

**TABLE 1:** Oligonucleotide primer sets for quantitative real-time PCR.

Name	Sequence (5′–3′)	Length (nt)	T_m_
Piezo1 F	ACTTTCCCATCAGCACTCGG	20	64
Piezo1 R	CCACGAAGTCCTTGAGACCC	20	64
TRPV2 F	TCGCTGTATGACCTGGCTTC	20	62
TRPV2 R	GCTCCAAAACGACCATTCGG	20	62
TRPV4 F	TCTCACCGCCTACTACCAGC	20	62
TRPV4 R	GTAGAGGGCTGCTGAGACGA	20	64
β-Actin F	CATGTACGTTGCTATCCAGGC	21	57.6
β-Actin R	CTCCTTAATGTCACGCACGAT	21	55.6

The relative expression of target genes was calculated by the 2^−ΔΔCT^, method. nt, nucleotides; T_m_, melting temperature.

### Immunofluorescence staining

hCMEC/D3 cells were fixed with 4% paraformaldehyde for 10 min at room temperature, then washed three times at room temperature using phosphate-buffered saline (PBS) and blocked with 5% normal goat serum for 30 min at room temperature. Cells were mixed with Piezo1 antibody (1:100; 15939-1-AP; Proteintech, China), TRPV4 (1:100; DF8624; Affinity Biosciences, China) and LAT1 (1:100; sc-374232; Santa Cruz Biotechnology, United States) overnight at 4 °C followed by 594-conjugated Goat Anti-Mouse IgG (H + L, 1:100; ABclonal, China) and Plus 488-Goat Anti-Rabbit Recombinant Secondary Antibody (H + L, 1:100; Proteintech, China) for 1 h at room temperature. Human brain tissue was obtained from one patient who underwent glioma resection surgery and written informed consent was provided by the patient. Brain tissue was fixed with 4% paraformaldehyde and 5 µm tissue slices were prepared for immunofluorescence staining as for hCMEC/D3 cells. The specificity of the antibodies for TRPV4 and Piezo1 have already been verified by the companies and the literature reports (Eisenhoffer et al., 2012). The immunofluorescence staining was analyzed using the Olympus BX53 positive fluorescence microscope (Olympus Corporation, Japan).

### Western blotting

The total protein was extracted using RIPA lysis buffer (NCM Biotech, China) containing proteinase inhibitors. The protein concentration was determined using bicinchoninic acid (BCA) kit (Epizyme Biotech, China). The samples were separated by sodium dodecyl-sulfate polyacrylamide gel electrophoresis (SDS-PAGE) and then transferred to polyvinylidene fluoride (PVDF) membranes by wet transfer. The membranes were incubated overnight at 4 °C with primary antibodies against the following proteins: Piezo1 (1:1,000; A23380; ABclonal, China), TRPV4 (1:1,000; A5660; ABclonal, China) and β-actin (1:5,000, AC026; ABclonal, China). After incubation with specific secondary antibodies, proteins were detected with an enhanced chemiluminescence kit (Millipore, Germany).

### Mechanical stress experiments

A custom-designed pressure system was employed to apply pressure stimulation. Briefly, the hCMEC/D3 cells were seeded on 6-well plates reached > 90% confluence before pressure exposure. The cells were exposed to mechanical pressure of 100 cm H_2_O at a frequency of 1 Hz (induced by pneumatic elements). Previous research from our lab confirmed that the pH, pO2, and pCO2 of the culture supernatants were not affected by the application of hydrostatic pressure at levels similar to those used in this study.

### Nitric oxide (NO) and ATP measurement

When cells cultured in 6-well plates reached > 90% confluence, they were stimulated with mechanical and pharmacological stimulation. Culture supernatants were obtained 2 min before and 1 min after stimulation.

NO concentration was determined with NO Colorimetric Assay Kit (Elabscience, Wuhan, China). Color developer (80 μL) was added to 160 μL sample, according to the manufacturer’s instructions. Samples were mixed and allowed to stand for 15 min and the absorbance values of each well at 550 nm measured following labelling with an enzyme marker. ATP concentration was determined with CellTiterGlo™ Luminescent Cell Viability Assay kit (Promega, Fitchburg, WI, United States). Samples were analyzed using a GloMax™ 20/20 luminometer (Promega).

### Chemicals

The following drugs were used: Yoda1 (MCE, China), Dooku1 (TargetMOI, China), GSK (MCE, China), HC067047 (TargetMOI, China), PPADS (MCE, China), and apyrase (Sigma-Aldrich, China); drugs were stored at −20 °C before use. Dilutions were made with deionized water or DMSO on the day of experiments. The final concentrations of DMSO were <0.1%. In all experimental groups, at least one parallel control experiment was performed in the presence of the carrier, which had no pharmacological effect on either tension or agonist-induced contraction of the preparations. Chemicals were used in concentrations shown to be effective in preliminary experiments or in concentrations used in previously published studies (Swain et al., 2020; Zhao et al., 2021).

### Statistical analyses

Continuous variables are expressed as mean ± SD. Differences between two groups were analyzed by paired or unpaired t-test. Excel™ (Microsoft, Redmond, WA, United States) and Prism 8.0.2 (GraphPad, San Diego, CA, United States) were used for analyses. Differences were considered statistically significant when p < 0.05.

## Results

### Mechanical stimulation evoked an [Ca^2+^]_i_ increase in hCMEC/D3 cells

To determine whether hCMEC/D3 cells are responsive to mechanical stimuli, we poked individual hCMEC/D3 cells with a glass micropipette (the tip of the pipette was indicated by the star in Figure 1A) and recorded the change in [Ca^2+^]_i_ using a Ca^2+^ imaging method. We found that poke stimulus evoked a rapid increase in [Ca^2+^]_i_ in stimulated cell (cell 1 in Figure 1B). Surprisingly, cells neighboring the stimulated cell also exhibited increased [Ca^2+^]_i_ (cell 2 and cell 3 in Figures 1A,B), and the [Ca^2+^]_i_ peak amplitude increase in neighboring cells reduced with distance from the stimulated cell (Figure 1B). These data suggest that elevated [Ca^2+^]_i_ in mechanically stimulated cells leads to release of a transmitter that acts on neighboring cells, thereby propagating a Ca^2+^ wave from one cell to the next.

hCMEC/D3 cells were also responsive to flow SS stimulation (Figures 1C,D); however, the amplitude of [Ca^2+^]_i_ increase induced by SS was relatively less than that provoked by poke stimulation (Figure 1B vs. 1D).

### mRNA and protein expression of mechano-sensing channels in cultured hCMEC/D3 cells

**FIGURE 2:**
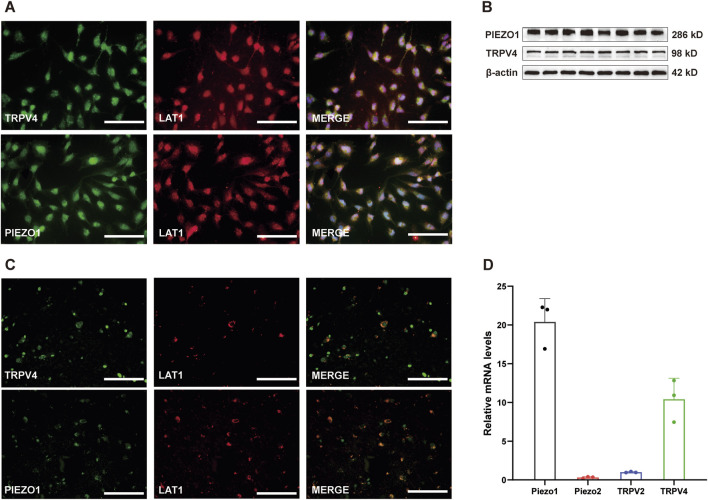
mRNA and protein expression of Piezo1 and TRPV4 in cultured hCMEC/D3 cells. **(A)** Immunofluorescence labeling showing Piezo1 and TRPV4 channels (green) are expressed on hCMEC/D3 cells. hCMEC/D3 cells were marked with the specific marker LAT1 (red), and their co-expression is shown in yellow. The nucleus marker (DAPI) is stained in blue. Scale bar = 100 μm. **(B)** Western blot showing Piezo1 and TRPV4 channels are expressed on hCMEC/D3 cells. **(C)** Immunofluorescence labeling showing Piezo1 and TRPV4 channels (green) are expressed on microvascular endothelial cells of human brain. The human brain microvascular endothelial cells were marked with the specific marker LAT1 (red), and their co-expression is shown in yellow. Scale bar = 100 μm. **(D)** Relative mRNA expression of mechano-sensing Piezo1, Piezo2, TRPV2, and TRPV4 channels. Total RNA was extracted from cultured hCMEC/D3 cells and subjected to RT-qPCR. The relative mRNA expression levels of Piezo1 and TRPV4 were higher than those of TRPV2 and Piezo2. Because the expression level of the observed channels was much lower than that of beta-actin, the mRNA expression of each channel was expressed as a relative level to that of TRPV2.

To examine which channels mediate the [Ca^2+^]_i_ increase triggered by mechanical stimuli in hCMEC/D3 cells, the expression levels of several mechanical sensing channels were first investigated in cultured hCMEC/D3 cells at the mRNA level by RT-qPCR. RT-qPCR experiments showed that the relative mRNA expression levels of PIEZO1 and TRPV4 were highest, while those of TRPV2 and Piezo2 were relatively low (Figure 2D). Accordingly, immunofluorescence and western bolt experiments revealed prominent staining for PIEZO1 and TRPV4 channels in cultured hCMEC/D3 cells (Figures 2A,B) stained with L-type amino acid transporter 1 (LAT1), one specific marker of HBMECs. To note, prominent staining of Piezo1 and TRPV4 channels were also found on microvascular endothelial cells of the human brain tissue (Figure 2C).

### Piezo1 and TRPV4 channels are functionally expressed in cultured hCMEC/D3 cells

**FIGURE 3:**
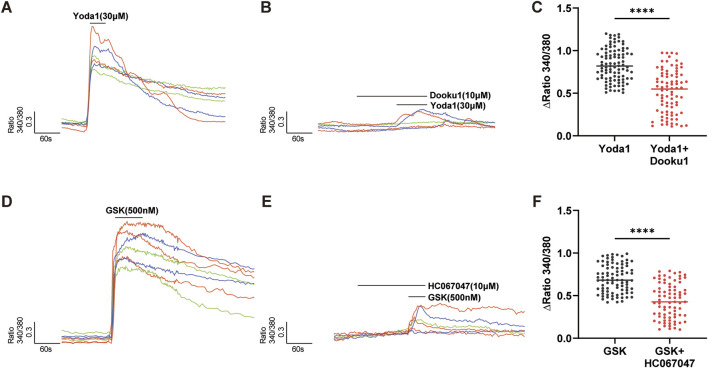
Piezo1 and TRPV4 channels are functional in hCMEC/D3 cells. **(A–C)** The specific agonist of Piezo1 channels, Yoda 1 (30 µM), evoked an increase in [Ca^2+^]_i_ **(A)**, which was significantly inhibited by pretreatment with Dooku1 (10 µM), a specific antagonist of Piezo1 channels **(B)**. Summary data are shown in **(C)**. **(D–F)** The specific agonist of TRPV4 channels, GSK1016790A (GSK, 500 nM), evoked an [Ca^2+^]_i_ increase **(D)** in hCMEC/D3 cells, which was significantly inhibited by pretreatment with HC067047 (10 µM), a specific antagonist of TRPV4 channels **(E)**. Summary data are shown in **(F)**. Numbers above each column indicate the numbers of cells. ****p < 0.0001.

Functional expression of Piezo1 and TRPV4 channels was examined by Ca^2+^ imaging using specific selective agonists (Figure 3). Treatment of cultured hCMEC/D3 cells (n = 180) with the Piezo1-specific agonist, Yoda 1 (30 µM), induced a significant increase in [Ca^2+^]_i_ (Figure 3A), and this effect of Yoda 1 on [Ca^2+^]_*i*_ was significantly diminished following pretreatment of cultured hCMEC/D3 cells with DooKu1 (10 µM), a Piezo1 antagonist (Evans et al., 2018) (Figures 3B,C). Application of GSK1016790A (GSK, 500 nM), a specific agonist of the TRPV4 channel, to cultured hCMEC/D3 cells (n = 165), induced a significant increase in [Ca^2+^]_I_ (Figure 3D), which was significantly blocked after pretreatment of cells with the TRPV4 specific antagonist, HC-067047 (1 µM) (Figures 3E,F). Together, the above findings suggest that Piezo1 and TRPV4 channels may be responsible for sensing mechanical stimuli in cultured hCMEC/D3 cells.

### Inhibition of Piezo1 and TRPV4 channels reduced poke-and SS-induced increases in [Ca^2+^]_i_

**FIGURE 4:**
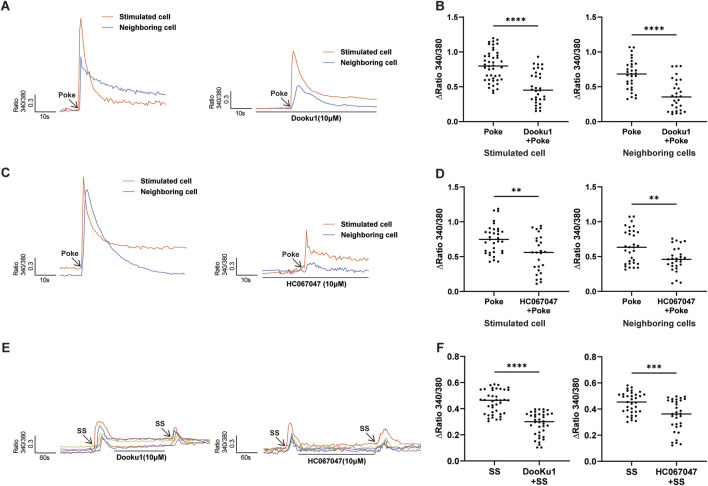
Inhibition of Piezo1 and TRPV4 channels attenuated poke-or SS-evoked increases in [Ca^2+^]_i_. **(A,B)** Typical traces **(A)** and summary data **(B)** showing that pretreatment with Piezo1 antagonist (Dooku1, 10 µM) reduced the increase in [Ca^2+^]_i_ induced by poke stimulation in stimulated and neighboring cells. **(C,D)** Typical traces **(C)** and summary data **(D)** show that pretreatment with TRPV4 antagonist (HC067047, 1 µM) reduced the increase in [Ca^2+^]_i_ induced by poke stimulation in stimulated and neighboring cells. **(E,F)** Typical traces **(E)** and summary data **(F)** indicating that pretreatment with Piezo1 (Dooku1, 10 µM) or TRPV4 (HC067047, 1 µM) antagonists reduced SS stimulation evoked increases in [Ca^2+^]_i_ in hCMEC/D3 cells. **p < 0.01, ***p < 0.001, ****p < 0.0001.

To investigate the involvement of Piezo1 and TRPV4 in hCMEC/D3 cell mechanical responses, the effects of Piezo1 and TRPV4 antagonists on poke- or SS-induced [Ca^2+^]_i_ were analyzed. As expected, poke-induced [Ca^2+^]_i_ increases was significantly reduced in the presence of Piezo1 (DooKu1, 10 μM) and TRPV4 (HC-067047, 1 μM) antagonists (Figures 4A–D) in both stimulated and neighboring cells. SS-induced increase in [Ca^2+^]_i_ were also significantly reduced in the presence of Piezo1 and TRPV4 antagonists (Figures 4E,F).

### Agonists of TRPV4 and Piezo1 channels evoked ATP secretion from cultured hCMEC/D3 cells

As demonstrated by the results presented in Figure 1, cells surrounding poked cells also showed increases in [Ca^2+^]_i_, suggesting the release of a transmitter from the poked cell that influences the surrounding cells in a paracrine manner.

**FIGURE 5:**
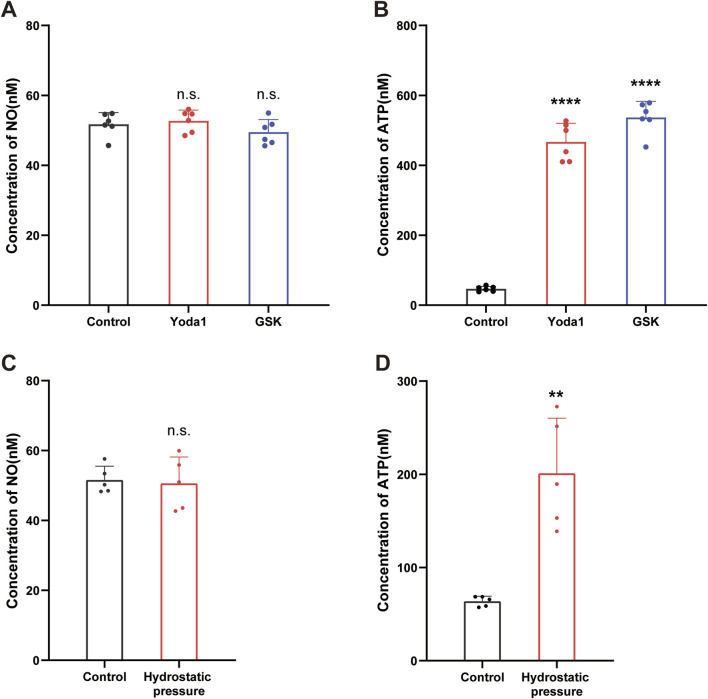
Activation of TRPV4 and Piezo1 channels evoked ATP release from cultured hCMEC/D3 cells. **(A,B)** Supernatant samples were collected 2 min before and 1 min after stimulation with Yoda-1 (30 µM) and GSK (500 nM) for 2 min and NO **(A)** and ATP **(B)** concentrations in the culture supernatants measured. **(C,D)** Supernatant samples were collected 2 min before and 1 min after stimulation with hydrostatic pressure (100 cmH_2_O for 1 min) and NO **(C)** and ATP **(D)** concentrations in the culture supernatants measured. Data presented in each graph are mean values from five culture dishes. **p < 0.01, ****p < 0.0001.

NO is considered a transmitter in cerebrovascular endothelial cells, and is released in response to [Ca^2+^]_i_ increase (Boedtkjer et al., 2011; Kawano et al., 2007; Lin et al., 2013). To determine whether NO was involved, we measured NO concentrations in culture supernatants before and after TRPV4 and piezo1 agonist stimulation. Unexpectedly, no significant change in NO concentration was detected after stimulation with the Piezo1 agonist, Yoda1 (30 μM), or the TRPV4 agonist, GSK (500 nM) (Figure 5A).

ATP is another transmitter involved in endothelial cell communication (Subauste, 2019). To assess ATP involvement in the observed phenomenon, we next measured ATP concentrations in culture supernatants before and after TRPV4 and Piezo1 agonist stimulation. As expected, application of the Piezo1 agonist, Yoda1 (30 µM), and the TRPV4 agonist GSK (500 nM), resulted in a significant increase in ATP concentration (Figure 5B).

To further confirm our findings, we conducted mechanical stress experiments. We measured the concentrations of ATP and NO in the culture supernatants before and after mechanical stimulation. Consistent with our pharmacological stimulation results, mechanical stimulation also led to an increase in ATP concentration (Figure 5D), while NO levels remained unchanged (Figure 5C).

### Release of ATP contributes to poke-induced [Ca^2+^]_i_ increase in both autocrine and paracrine manners

**FIGURE 6:**
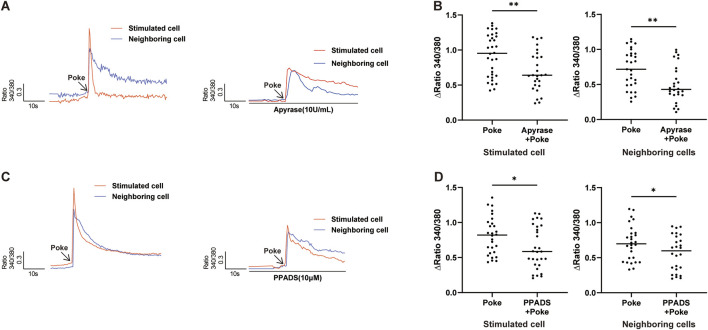
Release of ATP contributed to poke-induced [Ca^2+^]_i_ increase. **(A,B)** Typical traces **(A)** and summary data **(B)** demonstrating that application of apyrase (10 U/mL) to hydrolyze extracellular ATP reduced poke-induced [Ca^2+^]_I_ increase in stimulated and neighboring cells. **(C,D)** Typical traces **(C)** and summary data **(D)** showing that application of PPADS (10 μM), a common P2 receptor antagonist, reduced the poke-induced [Ca^2+^]_i_ increase in both stimulated and neighboring cells. Numbers above each bar indicate numbers of cells. *p < 0.05, **p < 0.01.

As previously demonstrated in peripheral vessel endothelial cells (Lillo et al., 2018), ATP also induced an increase in [Ca^2+^]_i_ in cultured hCMEC/D3 cells, and this ATP-evoked [Ca^2+^]_i_ increase could be reduced using the non-selective P2X and P2Y receptor blocker, pyridoxal-6-phenyl-2′,4′-disulfonic acid (PPADS). To investigate whether released ATP contribute to poke-induced [Ca^2+^]_i_ increase in an autocrine manner, the ATP-diphosphate hydrolase, apyrase, which can degrade extracellular ATP to ADP, and PPADS were applied. Pretreatment with apyrase (10 U/mL) and PPADS (10 μM) significantly inhibited the poke-induced increase in [Ca^2+^]_i_ in both poked and surrounding cells (Figure 6).

## Discussion

In the present study, we examined the expression of mechano-sensing TRPV4 and Piezo1 channels and their roles in the most widely-used and best-characterized human BMEC cell line, hCMEC/D3. Our major findings are: (1) Among mechano-sensing channels, TRPV4 and Piezo1 are more abundantly expressed than TRPV2 and Piezo2, which is consistent with previous reports of rodent (Vanlandewijck et al., 2018); (2) Activation of TRPV4 and Piezo1 channels contributes to mechanical stimulus-evoked [Ca^2+^]_i_ increase; (3) Activation of TRPV4 or Piezo1 channels induced ATP release from hCMEC/D3 cells; and (4) mechanical stimulus-induced responses in a single cell can elicit responses in surrounding cells by a paracrine mechanism involving ATP secretion. The expression and function of Piezo1 in brain endothelial cells have been studied in rodents (Harraz et al., 2022). To the authors’ knowledge, this is the first study to reveal the roles of Piezo1 and TRPV4 in mechano-transduction in hCMEC/D3 cells. Our results provide further evidence for an active role of HBMECs in mechano-transduction at the BBB.

Common *in vitro* methods of cellular mechano-stimulation include stretching cells with various custom-designed devices, exposing cells to high hydrostatic pressure, applying shear force to the cell surface, or poking cells with a glass micropipette (Dalghi et al., 2021; Neuhaus et al., 2020). In our study, we applied two mechanical modalities: poking and flow induced SS. We found that both stimuli can elicit cellular responses, manifesting as a transient [Ca^2+^]_i_ elevation in hCMEC/D3. Poking is reported to be approximately 20-fold better at increasing membrane tension than use of membrane suction for mechano-sensing channel activation (Coste et al., 2010; Guan et al., 2018; Kumar et al., 2020). In hCMEC/D3 cells, we also found that poke stimulus evoked a larger [Ca^2+^]_i_ increase than SS (Figure 1B vs. Figure 1D). Moreover, compared with SS, poke stimulus can be finely controlled (2 μm steps). Thus, although SS has been considered the standard physical stimulation method for HBMECs, poke was used as the mechanical stimulus in most of our experiments.

The BBB can respond to various mechanical forces, such as SS, pressure, and substrate stiffness. The role of SS in regulating BBB structure and function has been extensively studied (Garcia-Polite et al., 2017; Siddharthan et al., 2007; Wang et al., 2020). There is evidence that capillary-like SS promotes BBB function and facilitates the differentiation of vascular endothelial cells into a BBB phenotype (Rochfort et al., 2015), while high SS, due to systemic vascular disease or cerebral bypass graft, led to barrier disruption (Garcia-Polite et al., 2017). Matrix stiffness can regulate the local permeability of HBMECs (Yan et al., 2023); however, to our knowledge, no study has examined the molecular mechanism involved in mechano-sensing of HBMECs. The most important finding of our study is that Piezo1 and TRPV4 are involved in mechano-transduction in HBMECs. The supporting evidence includes: (1) TRPV4 and Piezo1 mRNA expression levels are the highest among mechano-sensing channels (Figure 1); (2) TRPV4 and Piezo1 protein expression were revealed both in HBMECs and in microvascular endothelial cells of human brain tissue (Figures 1, 2C); (3) TRPV4 and Piezo1 are functional in hCMEC/D3 cells, as demonstrated by the fact that TRPV4 or Piezo1 agonists evoked [Ca^2+^]_i_ increase; and, most importantly, (4) [Ca^2+^]_i_ increase evoked by mechanical stimulation was significantly blocked by selective antagonists of TRPV4 or Piezo1 (Figure 4). Therefore, the effects of mechanical force on BBB structure and function are likely mediated by opening of TRPV4 and Piezo1 channels and subsequent [Ca^2+^]_i_ increase.

Piezo1 channels have been shown to function as physiological sensors of blood flow (SS) in peripheral endothelial cells (Li et al., 2014). Endothelial Piezo1 mediates pressure-induced lung vascular hyperpermeability via disruption of adherens junctions (Friedrich et al., 2019). Further, TRPV4 serves as a mechanosensitive channel in endothelial cells that can mediate shear stress induced [Ca^2+^]_i_ increases, leading to the release of endothelial relaxing factors and dilation of small resistance arteries (Bagher et al., 2012; Mendoza et al., 2010; Sonkusare et al., 2012). Our data indicate that Piezo1 and TRPV4 channels serve as mechanosensors with similar roles in HBMECs to those described in peripheral endothelial cells (Caolo et al., 2020; Li et al., 2014). More interestingly, in one recent study, Piezo1 was reported to act as an upstream mediator of TRPV4 activation in endothelial cells in response to SS (Swain and Liddle, 2021); however, we did not test this possibility in HBMECs, as it is beyond our research interest.

HBMECs detect and respond to mechanical stimuli by triggering several downstream biochemical signaling pathways that control various cellular properties under physiological and pathophysiological conditions. Consistently, we found that activation of TRPV4 or Piezo1 channels with specific agonists induced the release of ATP (Figure 5B). This finding is also in agreement with recent reports showing that Piezo1 channel activation can induce ATP production in peripheral vascular endothelial cells (Jiang et al., 2023; Wang et al., 2016); however, unlike those studies, we found no evidence that Piezo1 activation is involved in NO release from hCMEC/D3 cells (Wang et al., 2016), suggesting that downstream signals in response to mechanical stimuli may differ between peripheral and cerebral endothelial cells.

Another important finding of our study is that mechanical stimulation of a single hCMEC/D3 cell can result in increased [Ca^2+^]_i_ in cells neighboring the stimulated cell, mediated by a paracrine ATP-secretion mechanism. Furthermore, we found that ATP release could further enhance the response to mechanical stimulation in an autocrine manner, as blocking the P2X and P2Y purinoceptors with PPADS or ATP hydrolase with apyrase significantly reduced the [Ca^2+^]_i_ increase in both stimulated and neighboring cells (Figure 6). Therefore, ATP may act as an amplifier of mechanical stimulation signal responses in hCMEC/D3 cells. Released ATP can activate both ionotropic (ligand-gated ion channels, i.e., the P2X family) and metabotropic (i.e., the P2Y family) receptors (Locovei et al., 2007; von Kügelgen and Hoffmann, 2016); however, we did not specifically detect which purinergic receptors are involved in these processes, and further clarification is warranted in future studies.

Limitations of our study: (1) only pharmacological approach has been used to block Piezo1 and TRPV4 channel activity, and no molecular approach such as siRNA knockdown has been applied to confirm our findings. (2) although SS may be a more physiologically relevant mechanical stimuli, however in most of our experiments, poke has been used. The reason is that poke stimuli could be finely controlled in our setup.

In summary, in this study we identified TRPV4 and Piezo1 channels as important mechanosensors in hCMEC/D3 cells. Further, our data reveal that paracrine and autocrine ATP release are important mechanisms involved in hCMEC/D3 cell mechanical sensing. In recent years, there has been considerable interest in the effects of mechanical force, such as cerebral vascular flow, in regulating the structure and function of the BBB under physiological and pathological conditions. Thus, revealing the pathological role of TRPV4 and Piezo1 in mechanical force alteration associated with BBB dysfunction has potential to facilitate development of new protective and restorative cerebral vascular interventional therapies.

## Data Availability

The raw data supporting the conclusions of this article will be made available by the authors, without undue reservation.
